# Editorial: Laparoscopic and Robotic Liver Surgery

**DOI:** 10.3389/fsurg.2022.854582

**Published:** 2022-02-23

**Authors:** Marcos V. Perini, Renato M. Lupinacci, Suk Kyun Hong

**Affiliations:** ^1^Department of Surgery at Austin Health, The University of Melbourne, Heidelberg, VIC, Australia; ^2^Hôpital Ambroise-Paré AP-HP, Université Paris Saclay, Boulogne-Billancourt, France; ^3^College of Medicine, Seoul National University, Seoul, South Korea

**Keywords:** laparoscopic liver surgery, robotic liver surgery, liver transplantation, cirrhosis, minimally invasive surgery (MIS), robotic 3D inspection system, HPB fellowship, hepatopancreatobiliary

Throughout the last two decades, several papers have confirmed the feasibility of minimally invasive liver surgery (MILS) and shown the advantages of MILS over open liver resection, especially regarding hospital stay and postoperative morbidity (1–4). However, the widespread use of MILS is not the reality yet, ranging from 10% in some countries (5) to up to 30% in others, depending on expertise, availability, and surgical volume (6). A recent survey has shown that, even in highly specialized European centers, MILS comprised about one third of all liver resections (2). These numbers are expected to increase further in the near future, once MILS have been incorporated as part of the general training program, especially in the HPB fellowship program.

Nowadays, most of MILS are performed laparoscopically, although the use of robotic liver surgery seems to be increasing even in challenging scenarios as shown by Golriz et al. and Solomonov et al. The robotic platform, apart from its ergonomic aspects, seems to facilitate the transition from open to MILS as has been demonstrated for other intra-abdominal procedures rather than liver resections. In addition, some complex interventions may be rendered easier using robotics, such as extended hepatectomies or resection in postero-superior segments.

Although MILS has a clear place in the most straight-forward liver resections (left lateral sectionectomy, lesions located in the antero-lateral liver, minor liver resection, and lesions not in close contact with major vascular structures), its use presents a major challenge when vascular resection and reconstruction are needed. Few authors have described portal vein resection/reconstruction however, the hepatic vein management by laparoscopy is still an area to be explored and tamed. The robotic approach, by overcoming the limitations of laparoscopy (unstable camera platform, two-dimensional vision, and ergonomically poor) is able to offer increased degrees of freedom, and a more precise range of movements (increased dexterity), which could be used during vascular reconstructions in liver surgery.

Higher costs, longer operative time, and robotic-CUSA unavailability are major drawbacks of the robotic when compared to the laparoscopic approach but the development of additional instruments for parenchymal transection might help to further increase the adoption of robotic MILS by most of the HPB high volume centers.

In high specialized HPB centers as shown by Hong et al., living related right donor hepatectomy has safely been done laparoscopically as a standard approach with better results in terms of blood loss, postoperative complications, and hospital stay in the expenses of a steep learning curve (7). On the other hand, robotic right donor hepatectomy (RRDH) surgery appears to circumvent the learning curve issue since the numbers of cases needed to achieve expertise are less than the ones needed for the laparoscopic counterpart. These same high-volume centers with expertise in the laparoscopic approach are also using the robotic platform to perform such a complex surgery, and have shown that although RRDH and laparoscopic right donor hepatectomy have the same complications rates and same advantages over the open approach, RRDH appears to have a less steep learning curve on the expenses of a cumbersome set up and the need of two senior surgeons (one in the console and the other one close to the patient).

Moreover, advances of technology (3D cameras, real time fluoroscopic guidance with indocyanine green as shown by He et al.) in the laparoscopic field have made subtle but important changes in the way surgeons were used to address liver surgery. Lastly, the comparable results in terms of blood loss and complication rates between MILS and open surgery and the benefits that the minimally invasive approach has over the open approach (in terms of wound complications, pain management, and hospital stay) has pushed surgeons to offer MILS as a standard of care in specific conditions (left lateral sectionectomy, small liver resections in antero-lateral segments). However, the near future of robotic surgery will probably force the HPB high volume specialized center to offer even more complex surgeries (left lateral donor hepatectomy, right donor hepatectomy), either by performing pure explant hepatectomies or by also offering the associated hybrid/robotic graft implantation in liver transplantation (8).

Robotic liver surgery, although in its youth, has arrived and is taking off. The fine balance between laparoscopic and robotic liver resection is yet to be defined in the years to come in HPB oncology and also in liver transplantation surgery.

## Author Contributions

MVP, RML, and SKH: writing and editing. All authors contributed to the article and approved the submitted version.

## Conflict of Interest

The authors declare that the research was conducted in the absence of any commercial or financial relationships that could be construed as a potential conflict of interest.

## Publisher's Note

All claims expressed in this article are solely those of the authors and do not necessarily represent those of their affiliated organizations, or those of the publisher, the editors and the reviewers. Any product that may be evaluated in this article, or claim that may be made by its manufacturer, is not guaranteed or endorsed by the publisher.
